# Excess Burden of Depression among HIV-Infected Persons Receiving Medical Care in the United States: Data from the Medical Monitoring Project and the Behavioral Risk Factor Surveillance System

**DOI:** 10.1371/journal.pone.0092842

**Published:** 2014-03-24

**Authors:** Ann N. Do, Eli S. Rosenberg, Patrick S. Sullivan, Linda Beer, Tara W. Strine, Jeffrey D. Schulden, Jennifer L. Fagan, Mark S. Freedman, Jacek Skarbinski

**Affiliations:** 1 Division of HIV/AIDS Prevention, Centers for Disease Control and Prevention, Atlanta, Georgia, United States of America; 2 Emory University Rollins School of Public Health, Atlanta, Georgia, United States of America; 3 Office of Public Health Preparedness and Response, Centers for Disease Control and Prevention, Atlanta, Georgia, United States of America; 4 National Institute on Drug Abuse, Rockville, Maryland, United States of America; University of Nebraska Medical Center, United States of America

## Abstract

**Background:**

With increased life expectancy for HIV-infected persons, there is concern regarding comorbid depression because of its common occurrence and association with behaviors that may facilitate HIV transmission. Our objectives were to estimate the prevalence of current depression among HIV-infected persons receiving care and assess the burden of major depression, relative to that in the general population.

**Methods and Findings:**

We used data from the Medical Monitoring Project (MMP) and the Behavioral Risk Factors Surveillance System (BRFSS). The eight-item Patient Health Questionnaire was used to identify depression. To assess the burden of major depression among HIV-infected persons receiving care, we compared the prevalence of current major depression between the MMP and BRFSS populations using stratified analyses that simultaneously controlled for gender and, in turn, each of the potentially confounding demographic factors of age, race/ethnicity, education, and income. Each unadjusted comparison was summarized as a prevalence ratio (PR), and each of the adjusted comparisons was summarized as a standardized prevalence ratio (SPR). Among HIV-infected persons receiving care, the prevalence of a current episode of major depression and other depression, respectively, was 12.4% (95% CI: 11.2, 13.7) and 13.2% (95% CI: 12.0%, 14.4%). Overall, the PR comparing the prevalence of current major depression between HIV-infected persons receiving care and the general population was 3.1. When controlling for gender and each of the factors age, race/ethnicity, and education, the SPR (3.3, 3.0, and 2.9, respectively) was similar to the PR. However, when controlling for gender and annual household income, the SPR decreased to 1.5.

**Conclusions:**

Depression remains a common comorbidity among HIV-infected persons. The overall excess burden among HIV-infected persons receiving care is about three-times that among the general population and is associated with differences in annual household income between the two populations. Relevant efforts are needed to reduce this burden.

## Introduction

With the advent of effective treatment and resultant increases in life expectancy, HIV infection has become a chronic condition. As with other chronic medical conditions, HIV infection is often complicated by comorbid depression [1], [2], and there are serious concerns regarding the adverse impacts of depression on the quality of life and the course of illness among those living with HIV. Depressed persons with HIV frequently become non-adherent with their treatment [3]–[5], which may lead to higher HIV viral loads, higher infectiousness, and poorer clinical outcomes. Some studies have suggested a more rapid progression to AIDS or an increase in mortality associated with depression among persons with HIV [6]–[11]. In addition, depression is associated with substance abuse [2], [12], which may contribute to reduced quality of life and increased participation in high-risk behaviors that may transmit HIV.

As the number of persons living with HIV increases, it becomes increasingly important to understand the extent of the burden of depression among these individuals, so that appropriate measures can be taken to alleviate this burden and maximize the effectiveness of HIV treatment and prevention. Although previous studies have reported estimates of depression among either HIV-infected persons or the general population, few have concurrently examined depression among both, using the same measure of depression. Findings from previous studies that have compared the prevalence of depression between HIV-infected and uninfected individuals have been mixed, but many of these studies were limited to groups with specific characteristics (e.g., men who have sex with men, injection drug users) and involved small convenience samples [13]–[22]. A meta-analysis combining data from 10 such studies found that the prevalence of depression was higher among HIV-infected persons, compared to uninfected persons [23]. However, no previous study – to our knowledge – has compared depression prevalence using large population-based samples of HIV-infected persons and the general population.

In this report, we address the question of whether HIV infection is associated with major depression by using population-based data to estimate the prevalence of current depression among persons infected with HIV and comparing it to the prevalence of depression among persons in the general population. We also assess the roles of major demographic factors in any excess burden of depression experienced by those with HIV infection.

## Methods

We describe the prevalence of depression in a nationally representative sample of HIV-infected persons receiving care using population-based data from the Medical Monitoring Project (MMP) and compare it to the prevalence of depression in a state-based probability sample of persons in the general population from the Behavioral Risk Factor Surveillance System (BRFSS).

### Medical Monitoring Project (MMP)

MMP is a supplemental HIV surveillance system designed to produce nationally representative estimates of behavioral and clinical characteristics of HIV-infected adults receiving medical care in the United States. MMP methods, including weighting procedures, have been described in detail elsewhere [24]–[26]. Briefly, MMP uses a three-stage, probability-proportional-to-size sampling method. U.S. states and territories were sampled, then eligible facilities, and finally, eligible individuals. Eligible facilities were those that provide outpatient HIV care, defined as the treatment and management of HIV disease, including monitoring CD4 and HIV viral load tests, or the prescription of antiretroviral medications. For the 2009 data collection cycle, eligible persons were HIV-infected adults age 18 years or older receiving medical care in participating facilities between January and April 2009. Data were collected through face-to-face interviews and medical record abstractions from June 2009 through May 2010. All sampled project areas participated in MMP. Participating areas included 16 states (California, Delaware, Florida, Georgia, Illinois, Indiana, Michigan, Mississippi, New Jersey, New York, North Carolina, Oregon, Pennsylvania, Texas, Virginia, and Washington), 6 separately-funded large metropolitan areas (Chicago, Houston, Los Angeles County, New York City, Philadelphia, and San Francisco) within 5 of the sampled states, and Puerto Rico. Data from MMP were weighted to produce estimates that represent all HIV-infected adults receiving care in the U.S.

### Ethics Statement

In accordance with the federal human subjects protection regulations at 45 *Code of Federal Regulations* 46.101c and 46.102d and with the *Guidelines for Defining Public Health Research and Public Health Non-Research* [27], [28], MMP was determined by the National Center for HIV, Viral Hepatitis, STD and TB Prevention’s Office of the Associate Director for Science at the Centers for Disease Control and Prevention (CDC) to be a non-research, public health surveillance activity used for disease control program or policy purposes. As such, MMP is not subject to human subjects regulations, including federal investigational review board (IRB) review. Nevertheless, participating project areas and facilities obtained local IRB approval to conduct MMP as required locally, and individual signed informed consents for participation in MMP were obtained as required locally. In addition, none of the authors of this manuscript has had access to any information that would directly identify individual persons on whom data were collected.

### Behavioral Risk Factor Surveillance System (BRFSS)

The BRFSS is a state-based surveillance system operated by state health departments in collaboration with the CDC. The objective of the BRFSS is to collect uniform, state-specific data on preventive health practices and risk behaviors that are linked to chronic diseases, injuries, and preventable infectious diseases in the adult population [29], [30]. Using a standardized questionnaire, trained interviewers conduct telephone interviews with individuals in a probability sample of households with telephones in the non-institutionalized U.S. adult population. Detailed BRFSS survey methodology, including weighting procedures, is described elsewhere [31].

In brief, the BRFSS questionnaire consists of three parts: core questions that are asked in all 50 states, the District of Columbia (D.C.), Puerto Rico, and the U.S. Virgin Islands; local questions that are added by the states; and optional supplemental modules, which are a series of questions on specific topics (for example, adult asthma history, intimate partner violence, and mental health)[32]. In 2006 and 2008, the state health departments, in collaboration with CDC and the Center for Mental Health Services, Substance Abuse and Mental Health Services Administration, collaborated on the implementation of the Anxiety and Depression Module, which consisted of questions about depression severity and lifetime diagnosis of anxiety and depression (Anxiety and Depression Module) [32]. During 2006 and 2008 combined, a total of 45 states, D.C., Puerto Rico, and the U.S. Virgin Islands implemented this module (29 states, D.C., Puerto Rico, and U.S. Virgin Islands in 2006; 16 states in 2008). For the 9 states that implemented the module during both 2006 and 2008 (Hawaii, Kansas, Louisiana, Maine, Mississippi, Nebraska, North Dakota, Vermont, and Washington), only the 2008 data were included in this analysis.

### Eight-item patient health questionnaire depression scale (PHQ-8)

The PHQ-8 consists of eight of the nine criteria on which the *Diagnostic and Statistical Manual of Mental Disorders, 4^th^ Edition (DSM-IV)* diagnosis of depressive disorders is based [33]. The ninth question in the *DSM-IV* assesses suicidal or self-injurious ideation and was omitted because not all interviewers were trained mental health providers. Research indicates that the deletion of this question has only a minor effect on scoring because active thoughts of self-harm are uncommon in the general population [34] and in primary care settings [35], [36]. The PHQ-8 has been shown to be comparable to the PHQ-9 [37], [38], which includes all nine DSM-IV criteria for depressive disorders and has demonstrated good psychometric properties in detecting major depression in non-psychiatric care settings. In a published review of validation studies, the PHQ-9 had reported values for sensitivity of 0.77 to 0.88, specificity of 0.88 to 0.94, internal reliability (Cronbach α) of 0.86 to 0.89, and external reliability (test-retest) of 0.84 in detecting major depressive disorders in a variety of care settings and patient populations [37]. Both the PHQ-8 and PHQ-9 have been used in clinical [39]–[44] and general population settings [45]–[48], and both have been used in self-administered [42]–[44], [48] and telephone-administered modes [45], [49]. The PHQ-9 has also been shown to be effective for detecting depressive symptoms in various racial and ethnic groups [44], [46], [49], [50].

For both MMP and BRFSS, participants were considered to have current major depression if, for “more than half the days” in the preceding two weeks, they met at least five of the eight criteria, including at least one of the following: 1) "little interest or pleasure in doing things" or 2) "feeling down, depressed, or hopeless." Participants were considered to have "other depression" if they experienced between two to four depressive symptoms for “more than half the days” in the preceding two weeks, with at least one being depressed mood or anhedonia. The PHQ-8 classification "other depression" comprises the DSM-IV categories of dysthymia and depressive disorder, not otherwise specified, which includes minor or subthreshold depression [33]. For the comparison of depression prevalence between MMP and BRFSS, we conducted standardized comparisons for major depression, which is more severe and disabling than other forms of depression.

### Analytical methods

For each of the two surveys, the distributions of demographic characteristics were first summarized with unweighted counts and weighted percentages with 95% confidence intervals (CI). Factors common to both surveillance systems were gender, age at the time of interview, race/ethnicity, and highest level of education attained. We additionally included time since HIV diagnosis and sexual orientation, which were only assessed in MMP. Next, the weighted prevalence and 95% CI of major, other, and any current depression among MMP participants were estimated overall and among the categories of demographic factors, analogous to published results for BRFSS in 2006 and 2008 [51], [52]. Comparisons between categories were made using the modified Rao-Scott χ^2^ Test [53].

To understand the degree to which the burden of major depression among HIV-infected adults receiving care is elevated compared to a probability sample drawn from the general U.S. population, we conducted stratified analyses that simultaneously controlled for gender and, in turn, one of the potentially confounding demographic factors of age, race/ethnicity, education, and income. The latter demographic factors were considered within gender categories because of the substantial differences in gender distribution between the two surveillance projects and the established relationship between gender and depression [2]. Within each stratum, we calculated the weighted prevalence and associated standard error (SE) of each depression outcome for both MMP and BRFSS. The weighted prevalences were compared between the two systems with prevalence ratios (PR). These stratum-specific estimates were then summarized with standardized prevalence ratios (SPRs) and their 95% CIs, utilizing the weighted prevalences and their SEs [54]. Thus, for each depression outcome, 4 SPRs were computed as the combinations of gender with age, race/ethnicity, education, or income. Together, these provided different perspectives of the excess in depression seen among HIV patients receiving care. This approach is an extension of the indirect method for calculating the age-adjusted standardized mortality ratios, in which the observed mortality rate in a population of interest is compared with an expected one derived from the age-specific mortality rates of a standard population [55]. With MMP as the population of interest and BRFSS as the standard population, factors known to be associated with depression were identified as key elements for standardization in our comparison, as explained above. To account for the complex survey designs, weighted analyses were conducted using PROC SURVEYFREQ in SAS 9.3 (SAS Institute, Cary, NC) for MMP data and SAS callable SUDAAN (RTI International, Research Triangle Park, NC) for BRFSS data. SPR calculations were performed in Microsoft Excel 2010 (Microsoft Corporation, Redmond, WA).

Although a high prevalence of depression has been observed among groups with increased risk for HIV, like men who have sex with men and injection drug users [12], [56]–[60], data were not available to adjust for these characteristics in our comparison of depression prevalence among MMP and BRFSS populations. However, we were able to determine the prevalence of depression among persons in the general population who reported one or more HIV risk behaviors, which might indicate that these individuals have an increased risk for HIV infection. To determine the presence of HIV risk behaviors, we analyzed responses to the following question, which was first included in the 2008 BRFSS core survey and was only directed to participants younger than 65 years of age: “I’m going to read you a list. When I’m done, please tell me if any of the situations apply to you. You do not need to tell me which one: 1) You have used intravenous drugs in the past year, 2) You have been treated for a sexually transmitted or venereal disease in the past year, 3) You have given or received money or drugs in exchange for sex in the past year, 4) You had anal sex without a condom in the past year. Do any of these situations apply to you?” [61]. The prevalence of major depression by gender and the presence of one or more HIV risk behaviors were calculated and summarized using unweighted counts and weighted percentages with 95% CI. Using the two-proportion Z test, statistical comparisons were made between the prevalence of major depression among persons in the general population with self-reported HIV risk behaviors and the prevalence of major depression in each of two other groups – persons in the general population who did not report such behaviors (BRFSS) and HIV-infected persons receiving care (MMP). These comparisons were made within gender categories to account for any gender-related difference in depression prevalence.

## Results

### Demographics

Among 4,217 MMP participants included in this analysis, 4,168 (98.7% of weighted sample) completed the PHQ-8 and comprised our analytic sample from MMP. As shown in Table 1, the largest proportions of the respondents who completed the PHQ-8 were male (71.2%), African-American (41.3%), and diagnosed with HIV at least 10 years previously (53.9%). A large proportion (59.1%) was age 45 or older, and only 14.2% was less than age 35. Although the largest proportion (49.3%) self-reported being heterosexual or straight, a considerable proportion (40.9%) reported being homosexual. The level of education was less than high school for 22.5%, and the annual household income was less than $10,000 for 34.5%.

**Table 1:** Self-reported characteristics of HIV-infected adults receiving medical care and general population adults in the United States who completed the Eight-item Patient Health Questionnaire depression scale — Medical Monitoring Project (MMP) 2009 and Behavioral Risk Factor Surveillance System (BRFSS), 2006 and 2008.

	MMP			BRFSS		
	*n sample*	*wgt. col %*	*(95% CI)*	*n sample*	*wgt. col %*	*(95% CI)*
**Overall**	4,168			235,067		
**Gender**						
Male	2,981	71.2	(68.1, 74.4)	89,842	48.6	(48.2, 49.1)
Female	1,123	27.1	(24.0, 30.3)	145,225	51.4	(50.9, 51.8)
Transgender*	64	1.6	(1.1, 2.1)	___	___	
**Age at interview**						
18–24	107	2.5	(1.8, 3.3)	9,944	12.6	(12.2, 13.0)
25–34	495	11.7	(10.4, 13.0)	27,086	18.7	(18.3, 19.1)
35–44	1,112	26.7	(25.3, 28.0)	39,440	20.2	(19.9, 20.6)
45–54	1,622	39.0	(37.2, 40.7)	49,623	19.0	(18.7, 19.3)
≥ 55	833	20.1	(18.6, 21.6)	107,265	29.5	(29.2, 29.8)
**Race/ethnicity^†^**						
Black/African American	1,718	41.3	(33.1, 49.6)	17,604	8.5	(8.3, 8.8)
Hispanic or Latino	874	19.2	(14.2, 24.2)	18,391	14.8	(14.4, 15.2)
White	1,383	34.7	(28.2, 41.3)	183,563	70.5	(70.1, 71.0)
Other	193	4.7	(3.7, 5.6)	13,528	6.1	(5.9, 6.4)
**Education**						
< High school (HS)	969	22.5	(19.9, 25.0)	21,463	10.8	(10.5, 11.1)
HS diploma or equivalent	1,149	26.8	(24.1, 29.6)	68,250	27.9	(27.5, 28.3)
> HS	2,050	50.7	(45.9, 55.5)	145,020	61.4	(60.9, 61.8)
**Annual Income**						
$0 to $9,999	1,458	34.5	(30.6, 38.3)	10,883	4.8	(4.6, 5.1)
$10,000 to $19,999	1,165	29.4	(27.6, 31.2)	26,540	11.2	(10.9, 11.5)
$20,000 to $49,999	881	23.0	(20.3, 25.8)	79,253	34.9	(34.5, 35.4)
$50,000+	491	13.1	(10.5, 15.6)	91,230	49.0	(48.5, 49.5)
**Time since HIV diagnosis**						
0 – 5 years	937	23.1	(21.2, 25.1)	___	___	
5 – 10 years	966	23.0	(21.5, 24.5)	___	___	
10+ years	2,261	53.9	(51.4, 56.3)	___	___	
**Sexual orientation**						
Homosexual, gay, or lesbian	1,679	40.9	(36.3, 45.6)	___	___	
Bisexual	342	8.2	(7.2, 9.2)	___	___	
Heterosexual or straight	2,080	49.3	(44.4, 54.3)	___	___	
Other	57	1.6	(0.9, 2.3)	___	___	

*Male-to-female or female-to-male; **^†^**mutually exclusive race/ethnicity categories.

wgt. col%  =  weighted column %; 95% CI = 95% confidence intervals.

Among 267,584 BRFSS participants who were offered participation in the Anxiety and Depression Module in 2006 or 2008, 235,067 (88.7% of weighted sample) completed the PHQ-8 and comprised the analytic sample from BRFSS. In contrast to the HIV-infected population receiving care, the largest proportion of the general population sample from BRFSS was female (51.4%) and white (70.5%). Less than half (48.5%) of the general population sample was age 45 or older, and a substantial proportion (31.3%) was younger than age 35. The level of education was less than high school for only 10.8%, and the annual household income was less than $10,000 for only 4.8% of the general population sample.

### Depression prevalence

Depression was common among HIV-infected persons receiving care, with PHQ-8 responses indicating an estimated 12.4% (95% CI: 11.2%, 13.7%) prevalence of current major depression and 13.2% (95% CI: 12.0%, 14.4%) of other depression, yielding 25.6% (95% CI: 23.8%, 27.4%) with any current depression. Statistically significant heterogeneity in the prevalence of major depression was seen by levels of gender (p<0.0001), age (p = 0.005), education (p = 0.001) and annual income (p<0.0001). Major depression was most common among female (17.0%) and transgender (17.5%) participants and among those between 35 and 44 years old (15%), with less than a high school education (16.2%), or with an annual income less than $10,000 (16.5%)(Table 2). No association was observed between major depression and race/ethnicity, time since HIV diagnosis, or sexual orientation.

**Table 2:** Weighted percentage of HIV-infected adults receiving medical care in the United States who met criteria for current depression*, by type of depression and selected self-reported characteristics — Medical Monitoring Project, 2009.

	Major depression			Other depression			Any current depression				
	*n*	*wgt. row %*	*(95% CI)*	*n*	*wgt. row %*	*(95% CI)*	*n*		*wgt. row %*		*(95% CI)*
**Total**	506	12.4	(11.2, 13.7)	535	13.2	(12.0, 14.4)	1,041	25.6		(23.8, 27.4)	
**Gender**											
Male	305	10.6	(9.2, 12.0)	367	12.7	(11.2, 14.2)	672	23.3		(21.4, 25.3)	
Female	189	17.0	(14.6, 19.3)	160	14.4	(12.4, 16.3)	349	31.3		(28.2, 34.4)	
Transgender^†^	12	17.5	(6.7, 28.4)	8	11.7	(3.7, 19.8)	20	29.3		(16.7, 39.9)	
**Age at interview**											
18–24	13	12.1	(3.9, 20.2)	11	12.0	(4.7, 19.4)	24	24.1		(12.8, 35.3)	
25–34	71	13.8	(10.3, 17.3)	66	13.7	(10.4, 17.0)	137	27.4		(23.1, 31.8)	
35–44	151	15.0	(12.8, 17.2)	146	13.3	(11.3, 15.2)	297	28.3		(25.0, 31.6)	
45–54	202	12.4	(10.3, 14.5)	216	14.0	(12.2, 15.9)	418	26.4		(23.8, 29.1)	
≥ 55	69	8.4	(6.5, 10.3)	96	11.1	(8.8, 13,4)	165	19.5		(16.7, 22.2)	
**Race/ethnicity**											
Black/African American	200	11.7	(9.8, 13.6)	252	15.0	(13.0, 17.1)	452	26.7		(24.0, 29.5)	
Hispanic or Latino	118	13.8	(11.0, 16.5)	111	12.8	(10.4, 15.1)	229	26.5		(23.1, 29.9)	
White	170	13.0	(11.0, 15.0)	145	11.0	(9.2, 12.7)	315	24.0		(21.3, 26.7)	
Other	18	9.6	(6.4, 12.8)	27	14.3	(7.4, 21.2)	45	23.9		(16.8, 31.1)	
**Education**											
< High school (HS)	153	16.2	(13.8, 18.7)	155	15.8	(13.4, 18.1)	308	32.0		(28.8, 35.1)	
HS diploma or equivalent	138	11.8	(9.7, 14.0)	167	15.3	(13.4, 17.3)	305	27.2		(24.6, 29.7)	
> HS	214	11.0	(9.4, 12.7)	213	10.8	(9.1, 12.5)	427	21.9		(19.4, 24.4)	
**Annual Income**											
$0 to $9,999	239	16.5	(14.1, 18.9)	232	17.0	(15.0, 19.0)	471	33.5		(30.4, 36.6)	
$10,000 to $19,999	137	12.6	(10.1, 15.1)	150	12.5	(10.0, 15.0)	287	25.1		(21.2, 29.0)	
$20,000 to $49,999	79	9.4	(7.4, 11.3)	98	11.7	(9.6, 13.8)	177	21.1		(18.0, 24.1)	
$50,000+	20	4.0	(2.5, 5.6)	26	5.8	(4.3, 7.3)	46	9.8		(7.7, 11.9)	
**Time since HIV diagnosis**											
0 – 5 years	117	11.9	(8.7, 15.1)	124	13.4	(10.6, 16.2)	241	25.3		(20.3, 30.3)	
5 – 10 years	113	11.9	(9.5, 14.2)	136	14.9	(12.7, 17.1)	249	26.8		(23.9, 29.7)	
10+ years	276	12.9	(11.2, 14.7)	274	12.3	(10.6, 13.9)	550	25.2		(22.7, 27.8)	
**Sexual orientation**											
Homosexual, gay, or lesbian	182	11.2	(9.6, 12.9)	187	11.6	(9.8, 13.4)	369	22.8		(20.2, 25.5)	
Bisexual	44	14.2	(10.0, 18.5)	55	16.5	(12.2, 20.7)	99	30.7		(23.9, 37.5)	
Heterosexual or straight	267	13.0	(11.2, 14.7)	286	14.0	(12.5, 15.6)	553	27.0		(24.6, 29.3)	
Other	11	18.1	(8.8, 27.3)	5	8.2	(0.2, 16.3)	16	26.3		(15.2, 37.4)	

*Based on 4,168 persons who completed the *Eight-item Patient Health Questionnaire* (PHQ-8) depression scale*;* ^†^Male-to-female or female-to-male.

wgt. row%  =  weighted row %; 95% CI = 95% confidence intervals.

Responses to the PHQ-8 were used to define “Major depression” and “Other depression” according to criteria from the *Diagnostic and Statistical Manual of Mental Disorders, 4^ th^ Edition*. Any depression is the presence of either major depression or other depression.

Based on BRFSS data, the prevalence of depression among general population adults was estimated to be 4.1% (95% CI: 3.9%, 4.2%) for current major depression and 5.1% (95% CI: 4.9%, 5.3%) for current other depression, yielding 9.1% (95% CI: 8.9%, 9.4%) for any current depression (Table S1), and variations across categories of gender, age, education, and annual income were similar to those seen among HIV-infected persons receiving care, as demonstrated by MMP data. In contrast to HIV-infected persons receiving care, however, statistically significant differences were seen across racial/ethnic groups, with a higher prevalence of major depression seen among non-Hispanic blacks (5.0%), Hispanics (4.7%), or non-Hispanic persons of other races (5.1%) than among white, non-Hispanic persons (3.7%) in the general population.

### Comparison of depression prevalence (MMP and BRFSS)

Overall, the unadjusted prevalence ratio of current major depression among HIV-infected adults receiving care, compared to those in the general population, was 3.1. Similar results were observed when comparing the prevalence of current major depression across the two populations by gender, with the prevalence ratio being 3.2 for men and 3.5 for women.

Stratified comparisons of current major depression between MMP and BRFSS are presented in Table 3. Standardized prevalence ratios (SPR) that controlled for gender and each of the factors age, race/ethnicity, and education, indicated a prevalence of current major depression in MMP that was approximately three-fold (3.3, 3.0, and 2.9, respectively) that seen in BRFSS. When controlling for gender and annual income, the estimated excess burden of major depression was lower, with an SPR of 1.5.

**Table 3:** Relative and standardized prevalence of major depression by gender and four demographic characteristics — Medical Monitoring Project (MMP), 2009 and Behavioral Risk Factor Surveillance System (BRFSS), 2006 and 2008.

	Male					Female					Standardized Prevalence Ratio	
	MMP		BRFSS			MMP		BRFSS				
	*wgt. %*	*(SE)*	*wgt. %*	*(SE)*	*PR*	*wgt. %*	*(SE)*	*wgt. %*	*(SE)*	*PR*	*SPR*	*(95% CI)*
**Age at interview**											3.33	(2.97, 3.74)
18–24	8.8	(5.4)	2.5	(0.4)	3.61	18.8	(7.0)	5.1	(0.5)	3.70		
25–34	10.1	(2.5)	3.7	(0.4)	2.75	21.1	(3.7)	4.7	(0.3)	4.46		
35–44	13.1	(1.7)	3.3	(0.3)	4.02	19.2	(2.2)	5.4	(0.3)	3.55		
45–54	11.5	(1.2)	4.0	(0.2)	2.87	15.3	(2.1)	5.8	(0.2)	2.64		
≥ 55	6.4	(1.2)	1.7	(0.2)	3.73	12.8	(2.4)	2.4	(0.1)	5.45		
**Race/ethnicity**											3.02	(2.68, 3.41)
Black/African American	9.3	(1.3)	3.8	(0.4)	2.46	14.9	(1.5)	6.1	(0.4)	2.45		
Hispanic or Latino	9.7	(1.4)	3.7	(0.5)	2.61	24.6	(3.4)	5.7	(0.4)	4.29		
White	12.5	(1.1)	3.0	(0.1)	4.19	16.0	(2.5)	4.4	(0.1)	3.63		
Other	7.0	(1.6)	4.4	(0.6)	1.57	22.2	(6.9)	6.0	(0.5)	3.72		
**Education**											2.88	(2.58, 3.21)
< High school (HS)	13.1	(1.8)	6.3	(0.6)	2.08	19.9	(2.1)	10.0	(0.6)	1.98		
HS diploma or equivalent	11.3	(1.3)	4.0	(0.3)	2.85	13.4	(1.8)	5.8	(0.2)	2.29		
> HS	9.5	(0.9)	2.3	(0.1)	4.04	16.9	(2.3)	3.5	(0.1)	4.78		
**Annual income**											1.45	(1.28, 1.65)
$0 to $9,999	13.5	(1.4)	11.8	(1.4)	1.15	21.0	(1.9)	16.0	(0.9)	1.31		
$10,000 to $19,999	11.7	(1.4)	8.0	(0.6)	1.46	15.2	(2.1)	10.6	(0.5)	1.43		
$20,000 to $49,999	9.5	(1.1)	3.8	(0.3)	2.52	8.7	(2.6)	5.0	(0.2)	1.75		
$50,000+	4.5	(0.9)	1.4	(0.1)	3.14	0.6	(0.6)	2.0	(0.10	0.30		

wgt. %  =  weighted %; SE  =  standard error; PR  =  prevalence ratio (unadjusted); SPR  =  standardized prevalence ratio; 95% CI = 95% confidence intervals.

Responses to the Eight-item Patient Health Questionnaire were used to define “major depression” according to criteria from the Diagnostic and Statistical Manual of Mental Disorders, 4^th^ Edition.

These relationships were similar for the two additional depression outcomes considered. For current other depression, the SPR controlling for gender with age was 2.9 (95% CI: 2.6, 3.2), with race/ethnicity 2.1 (95% CI: 1.9, 2.4), with education 2.4 (95% CI: 2.2, 2.6), with income 1.6 (95% CI: 1.4, 1.8). Considering the outcome of any current depression, the SPR controlling for gender with age was 3.1 (95% CI: 2.9, 3.3), with race/ethnicity 2.5 (95% CI: 2.3, 2.7), with education 2.6 (95% CI: 2.4, 2.8), with income 1.5 (95% CI: 1.4, 1.7).

### Sub-analysis of depression prevalence and HIV risk behaviors

Among 61,128 participants of the 2008 BRFSS survey <65 years of age who responded to the question about HIV risk behaviors, 1,475 (3.4% of weighted sample) reported they had one or more such behaviors in the preceding year. For male respondents, the prevalence of current major depression was 6.1% (95% CI: 3.9, 9.5) among 637 who reported having one or more HIV risk behaviors and 3.0% (95% CI: 2.6, 3.4) among 23,142 who did not report having such behaviors (p = 0.03). For female respondents, the prevalence of current major depression was 10.6% (95% CI: 7.6, 14.5) among 838 who reported having one or more HIV risk behaviors and 4.9% (95% CI: 4.4, 5.3) among 36,511 who did not report having such behaviors (p<0.01).

Men <65 years of age in the general population who reported one or more HIV risk behaviors had a lower prevalence of current major depression compared to men <65 years of age receiving care for HIV infection (6.1% vs. 10.6%, p<0.01). Women <65 years of age in the general population who reported one or more HIV risk behaviors had a lower prevalence of current major depression compared to women <65 years of age receiving care for HIV infection (10.6% vs. 17.0%, p<0.01).

## Discussion

In this report we present the first nationally representative data available on depression among HIV-infected adults receiving care in the U.S. since the HIV Cost and Services Utilization Study (HCSUS) was conducted during 1994–2000 [62], and the largest analysis of such data to date. Based on data from the 2009 MMP cycle, among HIV-infected persons receiving outpatient care in the U.S., more than a quarter – or over 100,000 – had symptoms of any depression, including about 12% – or over 50,000 – with symptoms that were consistent with a current major depressive episode. Given the chronic and episodic nature of depression, the full burden of this condition over time is likely greater than is demonstrated by our estimates of current depression. Although not directly comparable, the one-year prevalence of major depression estimated by HCSUS (22%) was nearly twice as high as our MMP estimate for current major depression [62].

The high burden of depression among HIV-infected persons is more apparent when compared with that among the general population. Based on our data, the prevalence of current major depression among HIV-infected persons receiving outpatient care was over three times that seen in a general population sample from BRFSS. Further, similar excesses in major depression prevalence were seen by gender, and the higher relative prevalences among HIV-infected persons appeared to be primarily related to lower annual household income. Although the prevalence ratio for current major depression changed somewhat when the two populations were standardized to account for differences in gender and either age (SPR = 3.3), race/ethnicity (SPR = 3.0), or educational attainment (SPR = 2.9), it changed dramatically when standardized for gender and annual household income level (SPR = 1.5). Lower socioeconomic status, particularly poverty, is known to be associated with HIV infection and with depression [63]–[67], and the nature of these relationships is likely complicated. For example, income may be an indicator of the degree of access to health care resources, which may affect HIV-related knowledge and behaviors and the general health status of individuals. Poor health status – manifesting as chronic illnesses or as conditions that might lead to such illnesses – may contribute to the risk for depression, and depression, in turn, may contribute to poorer health and lower socioeconomic status [65], [68], [69].

In comparing the depression prevalence between HIV-infected persons and the general population, it was important to recognize the differences in demographics between these two populations and the need to account for them, because certain demographic groups in which depression prevalence is known to be higher (persons with low socioeconomic status) or lower (men) are over-represented among HIV-infected persons [63], [70]. The SPRs calculated from MMP and BRFSS data provided a useful way to perform such a comparison and to assess whether there was an excess burden of major depression among HIV-infected persons receiving care beyond what would be expected based on their demographic composition alone. Based on our findings, socioeconomic factors, particularly annual household income, were important confounding factors that should be taken into account in such comparisons.

Although lower income accounted for much of the excess major depression among HIV patients, it did not fully account for it. An excess in depression prevalence remained, whether the prevalence ratio was adjusted for race/ethnicity, education, or household income. When standardized for annual household income, there was approximately a 50% excess depression among HIV patients. This finding is consistent with that from some previous studies, including a meta-analysis that reported that the odds of recent depression (within the past 1 to 6 months) were 2 times as great among persons with HIV, compared to those without HIV, and that sexual orientation did not account for the excess in major depression among those with HIV [23]. Further, our analysis of available data from BRFSS indicated that although the prevalence of current major depression was estimated to be higher among those with ≥1 risk factors for HIV than among those without such risk factors, it was still lower than the observed prevalence among MMP participants (who were living with HIV). Taken together, these observations suggest that, although some excess in major depression may be explained by circumstances related to risks for HIV acquisition, factors associated with having HIV – as a chronic illness – are also associated with higher prevalence of major depression.

It is notable that our estimate of the prevalence of current major depression among HIV patients is comparable to estimates of current depression associated with common chronic medical conditions such as diabetes mellitus (DM)(6.3%–13.3%, PHQ-8 algorithm for current major depression[71]) and cardiovascular disease (15.8% among persons ≥45 years, PHQ-8 score ≥10 [72]). The association between chronic medical illnesses and depression has been well-recognized, but the reasons for this are not fully understood. Multiple contributing factors – psychosocial, behavioral, and biological – are likely involved, and cross-sectional studies cannot establish their temporal relationships. Factors that might lead to comorbid depression include physical symptoms, side effects of medications, social stigma associated with the illness, and social isolation due to symptom severity or disability [3], [59], [68], [73]. Depression, in turn, might increase the risk for development of a chronic illness, or exacerbate an underlying chronic illness through its impact on behaviors (e.g., substance abuse, treatment non-adherence)[3], [68], [74], [75]. Immunologic and endocrinologic changes that are observed more commonly in people with than without depression might increase the risk for some chronic medical conditions or lead to poorer outcomes among those with a chronic medical condition [76]–[79]. Therefore, the importance of screening for and treatment of depression is increasingly being recognized, especially in the context of HIV or other chronic medical conditions [80]–[85].

Some limitations should be considered in interpreting the results in this report. Because MMP participants were all sampled through medical care providers, our results are representative of persons receiving care for HIV infection, but not all HIV-infected persons. It is not clear how the exclusion of those not receiving care for HIV would affect the prevalence estimates for depression, because depression might manifest as either increased care seeking [86] or not engaging in care [74], [87]. The use of the PHQ-8 might have led to the overestimation or underestimation of the prevalence of major depression. Overestimation might result from the fact that the PHQ-8 is not equivalent to a full diagnostic interview, and, therefore, might include depressive episodes caused by underlying mental or physical illnesses other than major depression [33]. The focus on identifying current (past 2 weeks) depression, however, might have led to underestimating the prevalence of major depression. Although helpful in minimizing recall bias, such a short time frame might not allow the detection of depression among individuals whose symptoms remit and recur episodically over time. Underestimation of the prevalence of depression might also result from the fact that individuals who have received care for their HIV infection might have received care that was effective in controlling their depression as well.

Several limitations might have affected the PRs and SPRs in our comparisons between MMP and BRFSS data. We were not able to standardize specifically for sexual orientation or substance abuse in our comparisons, which might have led to an overestimation of the PRs and SPRs. Nevertheless, our finding regarding major depression among general population adults with ≥1 HIV risk behaviors suggested that factors related to such behaviors did not fully account for the higher burden of major depression seen among HIV-infected persons receiving care. The difference in modes of administration between MMP (in-person) and BRFSS (telephone), in addition, might have contributed to underestimation of the PRs and SPRs. In a previous comparison of data from two national surveys, the depression prevalence estimate measured by the PHQ-8 was found to be higher with telephone administration than with in-person administration [88].

Limitations with BRFSS data are related to two main issues. First, the increase in the number households with cellular telephones only and in telephone number portability continue to decrease BRFSS response rates, reducing the precision of state estimates and potentially introducing bias [89]. Second, because not all states participated, estimates might not be generalizable to the entire U.S. adult population. Furthermore, due to the cross-sectional design of MMP and BRFSS, we were not able to make inferences regarding causality or temporality.

Despite the limitations of our analysis, it is clear that depression is an important comorbidity among HIV patients. Based on our results, about one in eight patients in care for HIV had current symptoms consistent with major depression, and about one in four had current symptoms of any type of depression. Using representative data sources for HIV-infected persons in care and the general population, the unadjusted prevalence of major depression among those in care for HIV is over three times that among the general population. This large relative difference in depression prevalence appeared to be primarily related to differences in annual household income. However, even after standardizing for income level, a smaller but important residual excess in depression burden remained, suggesting there might be additional factors contributing to this excess burden among HIV-infected persons who are receiving care.

Our findings also indicate that despite the availability of effective treatment for depression, many HIV-infected persons had symptoms consistent with major depression, raising concern about access to care and quality of care. Although some clinical guidelines exist for depression screening that are specific to HIV care settings [81], [84], it is not clear how widely adopted they are among HIV care providers, but there is evidence that many persons who are in care for HIV infection have depression that is not recognized and treated by their HIV care providers [90], [91]. Given the complexity of HIV treatment, HIV care providers may need training on issues related to the clinical management of depression in their patients, who may be taking multiple medications with various side effects and the potential for drug interactions. In addition, models for incorporating the management of depression within the HIV medical home have been developed and may be an effective approach to consider in ensuring appropriate care for HIV patients with depression in settings where access to mental health care is limited [92]. As interest grows in improving HIV prevention through antiretroviral treatment, it becomes increasingly important to recognize the role of comorbid depression in HIV infection and to closely integrate the screening for and treatment of depression with routine clinical management of HIV. Furthermore, because not all HIV-infected persons are in care, considerations should also be given to incorporating depression screening and treatment into HIV prevention programs, particularly those that address issues among those who are socioeconomically disadvantaged [93], [94]. Because of the potential detrimental effect of depression on HIV prevention and on the clinical outcomes of HIV-infected persons, effective treatment and follow-up over time are essential to the effective management of this chronic and disabling condition, which continues to affect many persons living with HIV in the U.S.

## Supporting Information

Table S1 **Weighted percentage of adults in the United States who meet criteria for current depression, by type of depression and selected characteristics - Behavioral Risk Factor Surveillance System, 2006 and 2008.** wgt. % =  weighted %; SE  =  standard error; PR  =  prevalence ratio (unadjusted); SPR  =  standardized prevalence ratio; 95% CI = 95% confidence intervals. Responses to the *Eight-item Patient Health Questionnaire* were used to define “major depression” according to criteria from the *Diagnostic and Statistical Manual of Mental Disorders, 4^th^ Edition*. Any depression is the presence of either major depression or other depression.
(DOCX)
