# Supplementary material for: Excess Burden of Depression among HIV-Infected Persons Receiving Medical Care in the United States: Data from the Medical Monitoring Project and the Behavioral Risk Factor Surveillance System
Source: PLoS One. 2014 Mar 24;9(3):e92842. doi: 10.1371/journal.pone.0092842 (PMC3963963; doi:10.1371/journal.pone.0092842)
Supplement: Table S1 — Weighted percentage of adults in the United States who meet criteria for current depression, by type of depression and selected characteristics - Behavioral Risk Factor Surveillance System, 2006 and 2008. wgt. % = weighted %; SE = standard error; PR = prevalence ratio (unadjusted); SPR = standardized prevalence ratio; 95% CI = 95% confidence intervals. Responses to the Eight-item Patient Health Questionnaire were used to define “major depression” according to criteria from the Diagnostic and Statistical Manual of Mental Disorders, 4th Edition. Any depression is the presence of either major depression or other depression. (DOCX) [file pone.0092842.s001.docx]

|  | **Major depression** | | | **Other depression** | | | **Any depression** | | |
| --- | --- | --- | --- | --- | --- | --- | --- | --- | --- |
|  | *n* | *wgt.row %* | *(95% CI)* | *n* | *wgt.row %* | *(95% CI)* | *n* | *wgt.row %* | *(95% CI)* |
| **Total** | 10,112 | 4.1 | (3.9-4.2) | 11,547 | 5.1 | (4.9-5.3) | 21,659 | 9.1 | (8.9-9.4) |
| **Gender** |  |  |  |  |  |  |  |  |  |
| Male | 2,978 | 3.3 | (3.0-3.5) | 4,165 | 4.8 | (4.5-5.1) | 7,143 | 8.0 | (7.6-8.4) |
| Female | 7,134 | 4.8 | (4.6-5.1) | 7,382 | 5.3 | (5.1-5.6) | 14,516 | 10.2 | (9.8-10.5) |
|  |  |  |  |  |  |  |  |  |  |
| **Age at interview** |  |  |  |  |  |  |  |  |  |
| 18-24 | 442 | 3.7 | (3.1-4.4) | 725 | 7.3 | (6.4-8.4) | 1,167 | 11.1 | (10.0-12.2) |
| 25-34 | 1,191 | 4.2 | (3.8-4.7) | 1,345 | 5.1 | (4.6-5.6) | 2,536 | 9.3 | (8.6-9.9) |
| 35-44 | 1,935 | 4.3 | (4.0-4.8) | 1,772 | 4.4 | (4.0-4.8) | 3,707 | 8.7 | (8.2-9.3) |
| 45-54 | 2,902 | 5.3 | (4.9-5.7) | 2,498 | 4.8 | (4.5-5.2) | 5,400 | 10.1 | (9.6-10.6) |
| ≥ 55 | 3,600 | 3.2 | (3.0-3.4) | 5,147 | 4.7 | (4.4-4.9) | 8,747 | 7.9 | (7.5-8.2) |
|  |  |  |  |  |  |  |  |  |  |
| **Race/ethnicity** |  |  |  |  |  |  |  |  |  |
| Black/African American | 981 | 5.0 | (4.5-5.6) | 1,387 | 7.9 | (7.0-8.8) | 2,368 | 12.9 | (11.9-14.0) |
| Hispanic or Latino | 1,063 | 4.7 | (4.1-5.4) | 1,367 | 7.0 | (6.2-7.8) | 2,430 | 11.7 | (10.8-12.7) |
| White | 7,194 | 3.7 | (3.6-3.9) | 7,885 | 4.2 | (4.1-4.4) | 15,049 | 8.0 | (7.7-8.2) |
| Other | 778 | 5.1 | (4.4-6.0) | 820 | 5.6 | (4.8-6.5) | 1,598 | 10.7 | (9.6-11.9) |
|  |  |  |  |  |  |  |  |  |  |
| **Education** |  |  |  |  |  |  |  |  |  |
| < High school (HS) | 1,938 | 8.1 | (7.4-9.0) | 2,118 | 9.2 | (8.4-10.1) | 4,056 | 17.4 | (16.2-18.6) |
| HS diploma or equivalent | 3,538 | 4.9 | (4.6-5.3) | 4,257 | 6.4 | (6.0-6.8) | 7,795 | 11.3 | (10.8-11.9) |
| > HS | 4,618 | 3.0 | (2.8-3.2) | 5,155 | 3.7 | (3.5-3.9) | 9,773 | 6.7 | (6.4-6.9) |
|  |  |  |  |  |  |  |  |  |  |
| **Annual Income** |  |  |  |  |  |  |  |  |  |
| $0 to $9,999 | 1,821 | 14.4 | (12.9-16.0) | 1,203 | 11.4 | (19.0-13.1) | 3,024 | 25.8 | (23.8-27.9) |
| $10,000 to $19,999 | 2,521 | 9.5 | (8.7-10.3) | 2,351 | 8.7 | (7.9-9.5) | 4,872 | 18.1 | (17.1-19.2) |
| $20,000 to $49,999 | 3,301 | 4.4 | (4.1-4.7) | 4,141 | 5.4 | (5.1-5.8) | 7,442 | 9.8 | (9.4-10.3) |
| $50,000+ | 1,500 | 1.7 | (1.5-1.9) | 2,402 | 3.1 | (2.8-3.3) | 3,902 | 4.8 | (4.5-5.1) |
|  |  |  |  |  |  |  |  |  |  |
